# Announcement: 19th Internation Symposium Industrial  Toxicology '99

**DOI:** 10.1155/MBD.1999.61

**Published:** 1999


					iN19th Internat cS( mposiu
DUSTRIAL I LOGY
June 16- 18, 1999
Bratislava, Slovak Republic
organized by
THE TOXICOLOGICAL SECTION OF THE SLOVAK SOCIETY
FOR INDUSTRIAL CHEMISTRY, BRATISLAVA
THE TOXICOLOGICAL CHEMISTRY SECTION
OF THE SLOVAK CHEMICAL SOCIETY,
SLOVAK ACADEMY OF SCIENCES, BRATISLAVA
IN COOPERATION WITH
THE FACULTY OF CHEMICAL TECHNOLOGY OF
THE SLOVAK UNIVERSITY OF TECHNOLOGY, BRATISLAVA
Contact e-mail address: agata@chtf.stuba.sk
Topics
1. Chemical substances and living organisms
2. Analysis and monitoring of toxic agencies in environment
3. Risk analysis assessment and management of chemical technologies and use of chemicals
4. Protection profilaxis and treatment of intoxication
5. Miscellaneous
Plenary lectures
M. Anke, Germany: The ultra trace element intake of nickel, cadmium, lead, vanadium,
titanium and strontium in Central Europe risk or normality?
A. Fargaov,, Slovak Republic: Metals in the environment
M. Herrchen, Germany: Analysis and management of technologies and chemicals by
combined use of the instruments of LCA and risk assessment
J. Lehotay, Slovak Republic: Trace analysis of some toxic organic compounds by HPLC
F. Lhuissier, France: Application of secondary ion mass spectrometry to the pollen as a
marker of atmospheric pollution
H.-P. Schmauder, Germany: Risk analysis of processes for manufacturing of biologically
degradable materials from growing raw materials
K.-W. Schramm, Germany: Biological in vitro investigation on industrial and environmental
samples
T. W. Schultz, USA.: Structure activity relationships: Quantitation of acute toxicity of
industrial organic chemicals
P. Tomasik, Poland: Metal toxicity why are we still alive?
61
Language
The Symposium language will be English.
Exhibition
A technical and scientific exhibition will be held in the hall of the Faculty of Chemical
Technology, Radlinskho 9, Bratislava during the symposium.
Registration fee
The conference fee should be paid to the account of the Slovak Society of Industrial Chemistry
ZSVTS, Slovenska sporitelna a.s., Bratislava, Obchodna 57, bank account: 111 341-
019/0900, constant symbol 0308, variable symbol: 8888. Acknowledgment of payment
together with an attached registration form should be dispatched to the symposium secretariat
before 15st March, 1999.
Societies members
Societies non members
Ph.D. students
Foreign participants
Until 15th March 1999
200 Sk (Kc)
500 Sk (Kc)
800 Sk (Kc)
250 US$
From 15th March 1999
500 Sk (Kc)
1 800 Sk (Kc)
000 Sk (Kc)
3O0 US$
The fee covers the attendance to all scientific sessions and undertakings, abstracts,
conference materials, coffee breaks.
Program
The program will include general sessions. All sessions will start with a plenary lectures (30-45
min) that will set the scene for each particular topic. Sessions will be scheduled in the morning
and afternoon with a lunch break including some time to visit the poster presentations. All other
lectures will last 15 min including discussion. Panels for a poster presentation (90 x 120 cm) will
be av,ilable, as welll as a slide projector and an overhead projector. Participants will receive the
conference program, proceedings and all materials at registration. Active participants will be
informed about the lecture (poster) insertion into the program under a separate letter.
Abstract submission
One or more abstracts of papers to be presented at the oral or poster sessions can be
submitted. The extended abstract should be 10 pacJes for plenary lectures, and 5 page for the
others, according to the Instructions for authors. The abstract, original plus onthe symposium
secretary.
Deadlines Notification of acceptation
Manuscript and Abstract
Early registration
15th March, 1999
15th March, 1999
end of April, 1999
Contact e-mail address: agata@chtf.stuba.sk
Prof. Agta Fragaovb
Department of Chemical Technology
Slovak University of Technology
Radlinskho 9
SK-812 37 Bratislava, Slovak Republic
62

				

